# RIPK1 is aberrantly expressed in multiple B-cell cancers and implicated in the underlying pathogenesis

**DOI:** 10.1007/s12672-023-00725-z

**Published:** 2023-07-18

**Authors:** Baoyu Wu, Jingyu Li, Han Wang, Jianguo Liu, Jiayong Li, Fang Sun, Dong chuan Feng

**Affiliations:** 1 Department of Pathology, Xuzhou Children’s Hospital, Xuzhou Medical University, 18 Sudi Road, Xuzhou, 221006 Jiangsu China; 2 Department of Pediatric Surgery, Xuzhou Children’s Hospital, Xuzhou Medical University, 18 Sudi Road, Xuzhou, 221006 Jiangsu China

**Keywords:** B-cell cancers, RIPK1, Necrostain-1, HOIPIN-1

## Abstract

According to the latest epidemiology of the US, B-cell cancers account for > 3% of all new cancer cases and > 80% of non-Hodgkin lymphomas. However, the disease-modifying small molecular drug suitable for most B-cell cancers is still lacking. RIPK1 (receptor-interacting serine/threonine-protein kinase 1) has been observed to be dysregulated and implicated in the pathogenesis of multiple solid cancers, of which, however, the roles in blood cancers are quite unclear. In our study, to identify multi-function targets for B-cell cancer treatment, we reanalyzed a public transcriptomic dataset from the database of Gene Expression Omnibus, which includes CD19^+^ B-cell populations from 6 normal donors and patients of 5 CLL, 10 FL, and 8 DLBCL. After overlapping three groups (CLL vs. normal, FL vs. normal, and DLBCL vs. normal) of differentially expressed genes (DEGs), we obtained 69 common DEGs, of which 3 were validated by real-time quantitative PCR, including RIPK3, IGSF3, TGFBI. Interestingly, we found that the loss function of RIPK1 significantly increases the proliferation and viability of GM12878 cells (a normal human B lymphocyte cell line). Consistently, overexpression of RIPK1 in TMD8 and U2932 cells effectively inhibited cell proliferation and growth. More importantly, modifying RIPK1 kinase activity by a small molecule (such as necrostain-1, HOIPIN-1, etc.) alters the cell growth status of B-cell lymphoma, showing that RIPK1 exhibits anti-tumor activity in the context of B-cell lymphoma. Taken together, we consider that RIPK1 may be a potential target in the clinical application of B-cell lymphoma (including CLL, DLBCL, and FL) treatment.

**Supplementary Information:**

The online version contains supplementary material available at 10.1007/s12672-023-00725-z.

## Introduction

The development of B lymphocytes derived from lymphoid progenitors is a complex cellular phenotypic change and differentiation progress, which involves a web of cell fate decisions in response to internal and external cues drives. During the maturation stage, B-cells with aberration in fate decisions should execute programmed cell death, but rare cells may escape from the quality control system to develop into cancer cells (the so-called B-cell cancers/lymphomas). According to the latest epidemiology of the US, B-cell cancers account for > 3% of all new cancer cases and > 80% of non-Hodgkin lymphomas (the most subcategory of hematological malignancies) [1, 2]. Importantly, the top three B-cell cancers, chronic lymphocytic leukemia (CLL), follicular lymphoma (FL), and diffusion large B-cell lymphoma (DLBCL), account for close to 70% of all non-Hodgkin lymphomas [3]. Although these three lymphomas originate from mature B-cells, they are distinct in the underlying pathogenesis and clinical presentation [1, 3]. Recently, Wong et al. constructed a BAFF (B-cell-activating factor) ligand-based chimeric antigen receptor T cell by targeting three receptors (BAFF-R, BCMA, and TACI), which was demonstrated to be effective in killing multiple B-cell cancers [4]. However, the disease-modifying small molecular drug that is suitable for most B-cell cancers is still lacking.

Receptor-interacting serine/threonine-protein kinase 1(RIPK1), a core component (scaffold function) of (tumor necrosis factor receptor 1) TNFR1 complex, serves as a vital cellular signaling hub that participates in TNF-mediated apoptosis and inflammatory pathways [5, 6]. Also, RIPK1 directly interacts with RIPK3 to promote the activation of necroptosis in a kinase activity-dependent manner [7]. In mouse models, RIPK1 depletion leads to multi-organ inflammation and abnormal cell death in animals [8, 9]. Meanwhile, RIPK1 deficiency in humans also causes severe immunodeficiency, intestinal inflammation, arthritis, etc., showing its evolutionary conserved functions among mammals [10, 11]. In the case of cancers, plenty of studies have recently demonstrated that RIPK1 plays a critical role in cancer immune checkpoint blockade (ICB) by modulating inflammation or immunity-related signaling pathway [12–14]. For instance, Cucolo et al. observed the expression level of RIPK1 is strongly associated with interferon-mediated resistance, and genetic deletion of RIPK1 in cancer cells leads to ICB failure by compromising chemokine secretion to decrease ARG1^+^ suppressive myeloid cells [14]. The vital functions of RIPK1 have been extensively investigated in solid tumors, such as hepatocellular carcinoma, soft-tissue sarcoma, melanoma, cervical Cancer, etc. [7, 15–17] However, whether and how RIPK1 is implicated in hematological malignancies (especially B-cell cancers) is still not fully explored.

To identify multi-function targets for B-cell cancer treatment, we reanalyzed a public transcriptomic dataset from the database of Gene Expression Omnibus, which includes CD19^+^ B-cell populations from 6 normal donors and patients of 5 CCL, 10 FL, and 8 DLBCL. After overlapping three groups (CLL vs. normal, FL vs. normal, and DLBCL vs. normal) of differentially expressed genes (DEGs), we obtained 69 common DEGs, of which 3 were validated using real-time quantitative PCR, including RIPK3, IGSF3, TGFBI, etc. Interestingly, we found that the knockdown of RIPK1 in GM12878 cells (a normal human B lymphocyte cell line) significantly increased cell proliferation and viability. Consistently, overexpression of RIPK1 in TMD8 and U2932 cells effectively inhibited cell proliferation and growth. More importantly, restoration of RIPK1 using HOIPIN-1 treatment significantly inhibited cell proliferation of TMD8 and U2932 cells. Altogether, RIPK1 is a promising target for B-cell lymphoma treatment.

## Methods and materials

### Human specimen collection and processing

In this study, 17 healthy donors and 55 B-cell lymphoma patients (including 14 children and 41 adults) were enrolled, consisting of 19 chronic lymphocytic leukemia (CLL), 15 diffuse large B-cell patients (DLBCL), and 21 follicular lymphomas (FL). The basic clinical information of patients was summarized in Table 1. The peripheral blood, lymph node, or tonsil tissues were collected (before pharmacological interventions) for the purification of CD19^+^ B-cells. The written informed consent was obtained from the participants or their families, and all the protocols used in our experiments were approved by the ethics committee of Xuzhou children’s hospital, Xuzhou Medical University.

**Table1:** basic clinical information of individuals involved in this study

Variable	CLL (n = 19)	DLBCL (n = 15)	FL (n = 21)	Overall (n = 55)
Age at baseline (years)				
Mean (sd)	54.6(20.2)	45.3(27.4)	45.1(24.8)	48.5(24.1)
Gender [n (%)]				
Male	7(36.8%)	5(33.3%)	9(42.9%)	21(38.2%)
Female	12(63.2%)	10(66.7%)	12(57.1%)	34(61.8%)
Weight(kg)				
Mean (sd)	58(9.6)	55.3(10.3)	55.4(11.9)	56.3(10.6)
Height(cm)				
Mean (sd)	159.5(8.2)	155.8(17.2)	152.9(19)	156(15.5)
Ann Arbor Stages				
I	2(10.5%)	5(33.3%)	17(81%)	24(43.6%)
II	7(36.8%)	7(46.7%)	4(19%)	18(32.7%)
III	7(36.8%)	2(13.3%)	(0%)	9(16.4%)
IV	2(10.5%)	1(6.7%)	(0%)	3(5.5%)
Missing	1(5.3%)	(0%)	(0%)	1(1.8%)
ECOG performance status				
0	7(35.6%)	5(58.6%)	9(48%)	21(45.5%)
1	12(64.4%)	10(41.4%)	12(52%)	34(54.5%)

Stages, classified using the modified Ann Arbor staging system

*CLL* chronic lymphocytic leukemia, *DLBCL* diffuse large B cell patients, *FL* follicular lymphomas

For the purification of CD19^+^ B-cells, the tissues of the lymph node or tonsil were mechanically dissociated and washed with PBS buffer containing 1% fetal bovine serum (FBS) and 2 mM EDTA. To remove the red blood cells, the grated tissues were incubated with lysis buffer containing 10 mM KHCO_3_, 150 mM NH_4_Cl, and 0.12 mM EDTA for 30 min at 4 °C. Then, the CD19^+^ B-cells were isolated from the treated tissues (consisting of PBMCs) using CD19 MicroBeads from Miltenyi Biotec company (Cat: 130-050-301, Shanghai, China) following the protocol provided by the manufacturer. The obtained CD19^+^ B-cell was subjected to total RNA isolation.

### Cell culture

GM12878 cells were purchased from Coriell Institute for Medical Research (NJ, USA), and cultured in RPMI-1640 containing 2 mM L-glutamine and 15%FBS. OCI-LY3 (ACC 761), RAJI (ACC 319), JEKO-1 (ACC 553), U2932 (ACC 633), WSU-FSCCL(ACC 612), and L428(ACC 197) cells were purchased from DSMZ-German Collection of Microorganisms and Cell Cultures (Braunschweig, GERMANY), and cultured in 85%RPMI-1640 plus 15%FBS. TMD8 (CBP600693) cells were purchased from COBIOER biosciences company (Nanjing China), and cultured in RPMI-1640 containing 10%FBS and 0.05 mM 2-mercaptoethanol. Human embryonic kidney (HEK) 293 T cells used for lentivirus packaging were purchased from American Type Culture Collection and were cultured in Gibco Dulbecco’s Modified Eagle Medium (DMEM) containing 10%FBS. All cells were maintained in a 5% CO_2_ incubator with an inside 37 °C condition.

### Construct stable cell lines

To construct RIPK1 knockdown and overexpression stable cell lines, home-made lentivirus vectors pLKO.1-puro (based on plasmid #8453 backbone from Addgene) and pLVX-M-puro (based on plasmid #125,839 from Addgene) were employed, respectively. For the packaging transgene lentivirus, HEK293T cells were co-transfected with helper plasmids psPAX.2 and pMD2.G with lentivirus backbone vector (carrying RIPK1 coding sequences or shRNA mimics) using Sinofection transfection reagent (STF02, shanghai, China) following manufacturer’s instructions. After 2 days of transfection, lentivirus supernatant was collected and filtered using a 0.45-mm filter. Next, target cells were directly infected using the obtained virus supernatant. After 12 h of infection, the virus-containing medium was replaced with a fresh culture medium and supplied with puromycin (ST551, Beyotime, Shanghai, China) at a dose of 1.0 μg/ml for positive selection. The oligos for the shRNA construct were listed as follows:shRIPK1_#1: Forward, 5ʹ—CCGG CAGC ACAA ATAC GAAC TTC AACT CGAG TTGA AGTT CGTA TTTG TGCT GTTT TTC—3ʹ; Reverse,5ʹ—AATT GAAA AACA GCAC AAAT ACGA ACTT CAAC TCGA GTTG AAGT TCGT ATTT GTGC TG—3ʹ shRIPK1_#2: Forward,5ʹ—CCGG AGGT CATG TTCT TTCA GCTT ACTC GAGT AAGC TGAA AGAA CATG ACCT TTTT TC-3ʹ; Reverse,5ʹ—AATT GAAA AAAG GTCA TGTT CTTT CAGC TTAC TCGA GTAA GCTG AAAG AACA TGAC CT—3ʹ; shRIPK1_#3: Forward,5ʹ—CCGG CCTT GTTG ATAA TGAC TTCC ACTC GAGT GGAA GTCA TTAT CAAC AAGG TTTT TC—3ʹ; Reverse,5ʹ—AATT GAAA AACC TTGT TGAT AATG ACTT CCAC TCGA GTGG AAGT CATT ATCA ACAA GG-3ʹ.

### Quantitative real-time PCR (qPCR)

The total RNA of B-cells was isolated using the RNAsimple kit from the Tiangen company (DP419, Beijing, China). Next, cDNA was synthesized from 1 μg total RNA using Quantscript Reversely transcription Kit (KR103, Tiangen, Beijing, China). Finally, qPCR was performed using FastFire qPCR PreMix (Cat: FP207, Tiangen, Beijing, China) on CFX Opus 96 Real-Time PCR System (Bio-rad, USA), and each sample was replicated at least three times. GAPDH served as the internal control, and the relative quantification of genes was calculated based on the ΔΔCt method. The oligos used in the study were listed in Table 2.

**Table 2:** Differentially expressed genes (DEGs) identified in this study

	ensembl_gene_id	baseMean	log2FoldChange	lfcSE	stat	pvalue	padj	symbol	description	bioType	EnterzgeneID	HGNC_ID	FL120	FL153
OSBPL5	ENSG00000021762	19.25425445	-4.651500455	1.025656934	− 4.535142601	5.76E-06	5.98E-05	OSBPL5	Oxysterol binding protein like 5 [Source:HGNC Symbol;Acc:HGNC:16392]	Protein_coding	114,879	HGNC:16,392	9	8
IPCEF1	ENSG00000074706	128.9721346	-4.338224913	0.696358542	− 6.229872477	4.67E-10	1.93E-08	IPCEF1	Interaction protein for cytohesin exchange factors 1 [Source:HGNC Symbol;Acc:HGNC:21204]	Protein_coding	26,034	HGNC:21,204	28	61
CST7	ENSG00000077984	35.17745193	-6.351241413	0.993360403	− 6.39369296	1.62E-10	7.71E-09	CST7	Cystatin F [Source:HGNC Symbol;Acc:HGNC:2479]	Protein_coding	8530	HGNC:2479	31	25
SMARCD3	ENSG00000082014	106.008591	-4.880376814	0.52035678	− 9.378905008	6.67E-21	4.50E-18	SMARCD3	SWI/SNF related, matrix associated, actin dependent regulator of chromatin, subfamily d, member 3 [Source:HGNC Symbol;Acc:HGNC:11108]	Protein_coding	6604	HGNC:11,108	85	195
SIGLEC1	ENSG00000088827	20.93313484	-5.631687887	1.204432053	− 4.675803732	2.93E-06	3.41E-05	SIGLEC1	Sialic acid binding Ig like lectin 1 [Source:HGNC Symbol;Acc:HGNC:11127]	Protein_coding	6614	HGNC:11,127	2	2
SOX10	ENSG00000100146	16.07498427	-5.687103505	1.001997868	− 5.675764075	1.38E-08	3.69E-07	SOX10	SRY-box transcription factor 10 [Source:HGNC Symbol;Acc:HGNC:11190]	Protein_coding	6663	HGNC:11,190	7	22
ADTRP	ENSG00000111863	57.63906662	-4.178468198	0.734422715	− 5.689459368	1.27E-08	3.42E-07	ADTRP	Androgen dependent TFPI regulating protein [Source:HGNC Symbol;Acc:HGNC:21214]	Protein_coding	84,830	HGNC:21,214	108	59
ROPN1B	ENSG00000114547	21.90928993	-5.654963228	0.981251932	− 5.763008503	8.26E-09	2.37E-07	ROPN1B	Rhophilin associated tail protein 1B [Source:HGNC Symbol;Acc:HGNC:31927]	Protein_coding	152,015	HGNC:31,927	23	104
PLCL1	ENSG00000115896	36.2190341	-4.425572473	0.776477649	− 5.699549087	1.20E-08	3.27E-07	PLCL1	P1hospholipase C like 1 (inactive) [Source:HGNC Symbol;Acc:HGNC:9063]	Protein_coding	5334	HGNC:9063	9	19
GRIN3B	ENSG00000116032	19.29138698	− 4.569998165	0.889077952	− 5.140154643	2.75E− 07	4.71E− 06	GRIN3B	Glutamate ionotropic receptor NMDA type subunit 3B [Source:HGNC Symbol;Acc:HGNC:16768]	Protein_coding	116,444	HGNC:16,768	7	25
PRRX1	ENSG00000116132	62.90985748	− 7.1904274	1.15242204	− 6.23940462	4.39E− 10	1.83E− 08	PRRX1	Paired related homeobox 1 [Source:HGNC Symbol;Acc:HGNC:9142]	Protein_coding	5396	HGNC:9142	13	74
TGFBI	ENSG00000120708	70.17753189	− 6.667978561	0.85002716	− 7.844430007	4.35E− 15	7.41E− 13	TGFBI	Transforming growth factor beta induced [Source:HGNC Symbol;Acc:HGNC:11771]	Protein_coding	7045	HGNC:11,771	59	67
EGR1	ENSG00000120738	3332.677443	5.425843533	0.536016317	10.12253426	4.39E− 24	4.82E− 21	EGR1	Early growth response 1 [Source:HGNC Symbol;Acc:HGNC:3238]	protein_coding	1958	HGNC:3238	420	774
PI3	ENSG00000124102	28.54999473	7.326915054	0.870335082	8.418499039	3.81E− 17	1.09E− 14	PI3	Peptidase inhibitor 3 [Source:HGNC Symbol;Acc:HGNC:8947]	Protein_coding	5266	HGNC:8947	1	0
FOSB	ENSG00000125740	6444.360908	5.409430431	0.710607435	7.612403372	2.69E− 14	3.59E− 12	FOSB	FosB proto− oncogene, AP− 1 transcription factor subunit [Source:HGNC Symbol;Acc:HGNC:3797]	Protein_coding	2354	HGNC:3797	2990	783
TMEM74B	ENSG00000125895	8.682578637	− 5.279355574	0.917646672	− 5.75314632	8.76E− 09	2.48E− 07	TMEM74B	Transmembrane protein 74B [Source:HGNC Symbol;Acc:HGNC:15893]	Protein_coding	55,321	HGNC:15,893	14	20
GLIS2	ENSG00000126603	19.83998958	− 6.486610596	0.994891921	− 6.519914836	7.03E− 11	3.74E− 09	GLIS2	GLIS family zinc finger 2 [Source:HGNC Symbol;Acc:HGNC:29450]	Protein_coding	84,662	HGNC:29,450	12	18
APOE	ENSG00000130203	228.4619596	− 6.174274732	0.74239677	− 8.316677794	9.05E− 17	2.35E− 14	APOE	Apolipoprotein E [Source:HGNC Symbol;Acc:HGNC:613]	protein_coding	348	HGNC:613	63	43
SPINK5	ENSG00000133710	84.04432459	9.16215216	0.863494168	10.61055477	2.66E− 26	5.84E− 23	SPINK5	Serine peptidase inhibitor Kazal type 5 [Source:HGNC Symbol;Acc:HGNC:15464]	Protein_coding	11,005	HGNC:15,464	0	0
EMP1	ENSG00000134531	209.6015354	5.383751607	0.620086528	8.682258633	3.88E− 18	1.40E− 15	EMP1	Epithelial membrane protein 1 [Source:HGNC Symbol;Acc:HGNC:3333]	Protein_coding	2012	HGNC:3333	5	9
AGT	ENSG00000135744	24.58099428	− 7.290746614	0.897751242	− 8.121121169	4.62E− 16	1.03E− 13	AGT	Angiotensinogen [Source:HGNC Symbol;Acc:HGNC:333]	Protein_coding	183	HGNC:333	25	79
CHRND	ENSG00000135902	6.666055491	− 4.897050912	1.038624615	− 4.71493824	2.42E− 06	2.92E− 05	CHRND	Cholinergic receptor nicotinic delta subunit [Source:HGNC Symbol;Acc:HGNC:1965]	Protein_coding	1144	HGNC:1965	3	8
IL1RN	ENSG00000136689	91.37593779	4.836036471	0.712422604	6.788156976	1.14E− 11	7.56E− 10	IL1RN	Interleukin 1 receptor antagonist [Source:HGNC Symbol;Acc:HGNC:6000]	Protein_coding	3557	HGNC:6000	7	15
IL36A	ENSG00000136694	29.47339375	8.772013712	1.242289579	7.061166623	1.65E− 12	1.34E− 10	IL36A	interleukin 36 alpha [Source:HGNC Symbol;Acc:HGNC:15562]	Protein_coding	27,179	HGNC:15,562	0	0
ENPP2	ENSG00000136960	215.7734377	− 5.943259452	0.926785233	− 6.412768829	1.43E− 10	6.93E− 09	ENPP2	Ectonucleotide pyrophosphatase/phosphodiesterase 2 [Source:HGNC Symbol;Acc:HGNC:3357]	Protein_coding	5168	HGNC:3357	20	16
RIPK1	ENSG00000137275	1672.093396	4.512926465	0.4626578	9.754350762	1.77E− 22	1.56E− 19	RIPK1	Receptor interacting serine/threonine kinase 1 [Source:HGNC Symbol;Acc:HGNC:10019]	Protein_coding	8737	HGNC:10,019	136	151
EVA1B	ENSG00000142694	67.31841674	− 4.542154309	0.835141546	− 5.438783794	5.36E− 08	1.18E− 06	EVA1B	Eva− 1 homolog B [Source:HGNC Symbol;Acc:HGNC:25558]	Protein_coding	55,194	HGNC:25,558	169	272
IGSF3	ENSG00000143061	128.6702029	− 6.900098858	0.795042876	− 8.678901561	4.00E− 18	1.43E− 15	IGSF3	Immunoglobulin superfamily member 3 [Source:HGNC Symbol;Acc:HGNC:5950]	Protein_coding	3321	HGNC:5950	126	200
LCN2	ENSG00000148346	126.7705225	10.30365243	1.003343621	10.26931573	9.69E− 25	1.31E− 21	LCN2	lipocalin 2 [Source:HGNC Symbol;Acc:HGNC:6526]	Protein_coding	3934	HGNC:6526	2	0
TCF7L1	ENSG00000152284	20.94621705	− 4.144226314	0.658261951	− 6.295709953	3.06E− 10	1.33E− 08	TCF7L1	transcription factor 7 like 1 [Source:HGNC Symbol;Acc:HGNC:11640]	Protein_coding	83,439	HGNC:11,640	25	31
VSIG4	ENSG00000155659	24.52393901	− 5.301813048	0.894588495	− 5.92653838	3.09E− 09	1.00E− 07	VSIG4	V− set and immunoglobulin domain containing 4 [Source:HGNC Symbol;Acc:HGNC:17032]	Protein_coding	11,326	HGNC:17,032	4	36
PDZD9	ENSG00000155714	7.852318706	− 5.625245103	0.940083273	− 5.983773207	2.18E− 09	7.38E− 08	PDZD9	PDZ domain containing 9 [Source:HGNC Symbol;Acc:HGNC:28740]	Protein_coding	255,762	HGNC:28,740	7	13
C1QC	ENSG00000159189	18.49822852	− 5.902796992	1.138299643	− 5.185626675	2.15E− 07	3.82E− 06	C1QC	Complement C1q C chain [Source:HGNC Symbol;Acc:HGNC:1245]	Protein_coding	714	HGNC:1245	3	5
CHRNB2	ENSG00000160716	73.1995986	− 4.388338218	0.645791563	− 6.795285772	1.08E− 11	7.23E− 10	CHRNB2	Cholinergic receptor nicotinic beta 2 subunit [Source:HGNC Symbol;Acc:HGNC:1962]	Protein_coding	1141	HGNC:1962	62	116
GRIN2C	ENSG00000161509	23.37444899	− 4.45191811	0.71189064	− 6.253654508	4.01E− 10	1.69E− 08	GRIN2C	Glutamate ionotropic receptor NMDA type subunit 2C [Source:HGNC Symbol;Acc:HGNC:4587]	Protein_coding	2905	HGNC:4587	46	47
VCAM1	ENSG00000162692	172.2405269	− 10.09135785	1.211972153	− 8.326394152	8.33E− 17	2.20E− 14	VCAM1	Vascular cell adhesion molecule 1 [Source:HGNC Symbol;Acc:HGNC:12663]	Protein_coding	7412	HGNC:12,663	14	23
SPRR3	ENSG00000163209	654.3400278	13.25004165	0.959650345	13.80715561	2.31E− 43	8.10E− 39	SPRR3	Small proline rich protein 3 [Source:HGNC Symbol;Acc:HGNC:11268]	Protein_coding	6707	HGNC:11,268	1	0
SPRR2D	ENSG00000163216	63.48325347	9.594372884	0.952101213	10.07705143	6.98E− 24	7.42E− 21	SPRR2D	Small proline rich protein 2D [Source:HGNC Symbol;Acc:HGNC:11264]	Protein_coding	6703	HGNC:11,264	0	0
CTLA4	ENSG00000163599	119.3098047	− 4.501004044	0.634188121	− 7.097269556	1.27E− 12	1.07E− 10	CTLA4	Cytotoxic T− lymphocyte associated protein 4 [Source:HGNC Symbol;Acc:HGNC:2505]	Protein_coding	1493	HGNC:2505	75	60
RNASE7	ENSG00000165799	33.24187915	8.076245733	0.973362728	8.297262161	1.07E− 16	2.69E− 14	RNASE7	Ribonuclease A family member 7 [Source:HGNC Symbol;Acc:HGNC:19278]	Protein_coding	84,659	HGNC:19,278	0	0
C15orf48	ENSG00000166920	63.70796296	5.741708677	0.861358532	6.665875433	2.63E− 11	1.57E− 09	C15orf48	Chromosome 15 open reading frame 48 [Source:HGNC Symbol;Acc:HGNC:29898]	Protein_coding	84,419	HGNC:29,898	15	4
CXCL8	ENSG00000169429	50.83518009	4.654179159	0.927750707	5.016626908	5.26E− 07	8.12E− 06	CXCL8	C− X− C motif chemokine ligand 8 [Source:HGNC Symbol;Acc:HGNC:6025]	Protein_coding	3576	HGNC:6025	44	1
CRCT1	ENSG00000169509	9.118403483	7.066510361	1.061193459	6.659021782	2.76E− 11	1.63E− 09	CRCT1	Cysteine rich C− terminal 1 [Source:HGNC Symbol;Acc:HGNC:29875]	Protein_coding	54,544	HGNC:29,875	1	0
FOS	ENSG00000170345	10,877.02658	4.152074154	0.773077814	5.370836003	7.84E− 08	1.61E− 06	FOS	Fos proto− oncogene, AP− 1 transcription factor subunit [Source:HGNC Symbol;Acc:HGNC:3796]	Protein_coding	2353	HGNC:3796	12,266	2291
KRT78	ENSG00000170423	90.42515389	9.523857489	0.994836317	9.573290925	1.04E− 21	8.08E− 19	KRT78	Keratin 78 [Source:HGNC Symbol;Acc:HGNC:28926]	Protein_coding	196,374	HGNC:28,926	1	0
GIMAP8	ENSG00000171115	66.92925053	− 6.195656893	0.942664567	− 6.572493662	4.95E− 11	2.75E− 09	GIMAP8	GTPase, IMAP family member 8 [Source:HGNC Symbol;Acc:HGNC:21792]	Protein_coding	155,038	HGNC:21,792	19	28
KRT13	ENSG00000171401	214.4699512	11.06210891	1.003617447	11.02223655	2.99E− 28	1.16E− 24	KRT13	Keratin 13 [Source:HGNC Symbol;Acc:HGNC:6415]	Protein_coding	3860	HGNC:6415	0	0
ADCY5	ENSG00000173175	39.95038232	− 4.736502201	0.832071993	− 5.692418731	1.25E− 08	3.38E− 07	ADCY5	Adenylate cyclase 5 [Source:HGNC Symbol;Acc:HGNC:236]	Protein_coding	111	HGNC:236	23	17
C1QA	ENSG00000173372	31.89538865	− 7.174670906	1.171308337	− 6.125347767	9.05E− 10	3.43E− 08	C1QA	Complement C1q A chain [Source:HGNC Symbol;Acc:HGNC:1241]	Protein_coding	712	HGNC:1241	5	4
ODF3	ENSG00000177947	16.26498334	− 6.19547321	0.977790294	− 6.336198313	2.36E− 10	1.07E− 08	ODF3	Outer dense fiber of sperm tails 3 [Source:HGNC Symbol;Acc:HGNC:19905]	Protein_coding	113,746	HGNC:19,905	12	39
TMPRSS9	ENSG00000178297	15.28991125	− 6.115447494	1.157500743	− 5.28332058	1.27E− 07	2.44E− 06	TMPRSS9	Transmembrane serine protease 9 [Source:HGNC Symbol;Acc:HGNC:30079]	Protein_coding	360,200	HGNC:30,079	12	20
SRPK3	ENSG00000184343	27.1006862	− 4.770906899	0.84759279	− 5.628772393	1.81E− 08	4.67E− 07	SRPK3	SRSF protein kinase 3 [Source:HGNC Symbol;Acc:HGNC:11402]	Protein_coding	26,576	HGNC:11,402	15	18
TMPRSS11B	ENSG00000185873	157.6573824	10.61799964	0.972338439	10.92006571	9.24E− 28	2.95E− 24	TMPRSS11B	Transmembrane serine protease 11B [Source:HGNC Symbol;Acc:HGNC:25398]	Protein_coding	132,724	HGNC:25,398	0	0
ZACN	ENSG00000186919	375.6300258	− 4.405791529	0.555732804	− 7.927895386	2.23E− 15	4.18E− 13	ZACN	Zinc activated ion channel [Source:HGNC Symbol;Acc:HGNC:29504]	Protein_coding	353,174	HGNC:29,504	788	867
SIGLEC15	ENSG00000197046	19.15500342	− 5.018354002	0.817447971	− 6.139050045	8.30E− 10	3.17E− 08	SIGLEC15	Sialic acid binding Ig like lectin 15 [Source:HGNC Symbol;Acc:HGNC:27596]	Protein_coding	284,266	HGNC:27,596	24	37
LYPD2	ENSG00000197353	13.57843739	6.519775246	1.152197754	5.658555767	1.53E− 08	4.03E− 07	LYPD2	LY6/PLAUR domain containing 2 [Source:HGNC Symbol;Acc:HGNC:25215]	Protein_coding	137,797	HGNC:25,215	0	0
RNA5S1	ENSG00000199352	236.5579431	5.710386043	0.594844183	9.599801442	8.01E− 22	6.39E− 19	RNA5S1	RNA, 5S ribosomal 1 [Source:HGNC Symbol;Acc:HGNC:34362]	rRNA	100,169,751	HGNC:34,362	18	25
SPDYE2	ENSG00000205238	51.23972902	− 4.400601067	0.839793358	− 5.240099872	1.60E− 07	2.98E− 06	SPDYE2	Speedy/RINGO cell cycle regulator family member E2 [Source:HGNC Symbol;Acc:HGNC:33841]	Protein_coding	441,273	HGNC:33,841	35	59
SMTNL1	ENSG00000214872	12.78844262	− 6.340824592	1.23594115	− 5.130361256	2.89E− 07	4.91E− 06	SMTNL1	Smoothelin like 1 [Source:HGNC Symbol;Acc:HGNC:32394]	Protein_coding	219,537	HGNC:32,394	20	19
CERS1	ENSG00000223802	6.63121223	− 4.358422288	0.975188259	− 4.469313745	7.85E− 06	7.71E− 05	CERS1	Ceramide synthase 1 [Source:HGNC Symbol;Acc:HGNC:14253]	Protein_coding	10,715	HGNC:14,253	3	7
CACTIN− AS1	ENSG00000226800	13.34614807	− 5.424277759	0.919272321	− 5.900621214	3.62E− 09	1.15E− 07	CACTIN− AS1	CACTIN antisense RNA 1 [Source:HGNC Symbol;Acc:HGNC:31391]	lncRNA	404,665	HGNC:31,391	26	31
EHBP1− AS1	ENSG00000231609	10.45641408	− 4.534220747	0.850447211	− 5.331572244	9.74E− 08	1.95E− 06	EHBP1− AS1	EHBP1 antisense RNA 1 [Source:HGNC Symbol;Acc:HGNC:55766]	lncRNA	100,132,215	HGNC:55,766	14	22
SNORD13	ENSG00000239039	22.66583357	4.408655214	0.605682338	7.278824124	3.37E− 13	3.38E− 11	SNORD13	Small nucleolar RNA, C/D box 13 [Source:HGNC Symbol;Acc:HGNC:32711]	snoRNA	692,084	HGNC:32,711	7	7
SPRR2A	ENSG00000241794	108.697552	10.94894654	0.933412788	11.73001557	8.94E− 32	7.85E− 28	SPRR2A	Small proline rich protein 2A [Source:HGNC Symbol;Acc:HGNC:11261]	Protein_coding	6700	HGNC:11,261	0	0
LINC00861	ENSG00000245164	153.6992183	− 5.401263923	0.78988884	− 6.838005104	8.03E− 12	5.57E− 10	LINC00861	Long intergenic non− protein coding RNA 861 [Source:HGNC Symbol;Acc:HGNC:45133]	lncRNA	100,130,231	HGNC:45,133	17	134
RPL36A− HNRNPH2	ENSG00000257529	119.7391129	5.188862458	0.699613038	7.416760661	1.20E− 13	1.35E− 11	RPL36A− HNRNPH2	RPL36A− HNRNPH2 readthrough [Source:HGNC Symbol;Acc:HGNC:48349]	Protein_coding	100,529,097	HGNC:48,349	26	14
NDUFC2− KCTD14	ENSG00000259112	220.1331784	4.812823629	0.619517404	7.768665731	7.93E− 15	1.26E− 12	NDUFC2− KCTD14	NDUFC2− KCTD14 readthrough [Source:HGNC Symbol;Acc:HGNC:42956]	Protein_coding	100,532,726	HGNC:42,956	26	48
TEX52	ENSG00000283297	22.02449776	− 4.264995461	0.755919471	− 5.642129389	1.68E− 08	4.35E− 07	TEX52	Testis expressed 52 [Source:HGNC Symbol;Acc:HGNC:53643]	Protein_coding	101,929,469	HGNC:53,643	13	30

	FL174	FL202	FL238	FL255	FL301	FL303	FL313	FL3A145	TS060911A	TS072111A	TS072611B	TS081111A	TS121511A	TS121511B
OSBPL5	29	12	27	391	11	6	272	26	2	0	0	2	0	1
IPCEF1	298	104	120	1659	78	46	252	177	10	5	6	7	7	8
CST7	60	10	79	334	65	24	114	45	1	0	0	1	0	1
SMARCD3	245	40	217	588	250	182	259	205	3	4	4	10	2	0
SIGLEC1	82	4	2	139	12	70	36	123	0	0	2	1	0	0
SOX10	21	15	30	97	19	20	91	17	0	0	0	2	0	0
ADTRP	59	44	50	303	54	12	450	79	4	0	0	7	5	4
ROPN1B	50	28	4	31	69	14	58	55	0	0	0	1	0	2
PLCL1	57	37	44	340	25	23	161	53	2	1	0	3	3	2
GRIN3B	42	13	41	163	27	39	43	9	0	0	0	4	0	1
PRRX1	26	34	77	693	81	12	381	28	0	0	1	2	0	0
TGFBI	149	19	51	474	94	184	385	97	0	1	2	0	0	2
EGR1	138	76	157	445	58	118	352	129	8039	3660	8452	491	11,222	8316
PI3	0	0	1	1	1	0	2	0	54	22	12	35	185	16
FOSB	151	96	45	462	176	165	149	130	10,881	9050	17,377	3008	11,987	24,850
TMEM74B	9	8	12	31	15	11	19	39	0	0	0	1	0	0
GLIS2	45	16	28	168	31	20	68	25	0	0	0	1	0	0
APOE	377	82	341	2625	128	160	975	428	4	3	2	3	3	7
SPINK5	1	0	0	1	0	0	2	1	115	26	210	187	365	46
EMP1	14	10	19	42	4	10	35	20	220	31	295	649	862	92
AGT	71	10	74	52	41	49	46	78	0	0	0	0	0	0
CHRND	12	6	13	59	5	4	16	16	0	0	0	1	0	0
IL1RN	1	7	1	2	5	2	55	9	109	73	142	205	360	69
IL36A	0	0	0	0	0	0	1	0	13	1	12	167	74	6
ENPP2	133	72	246	1597	43	22	2602	200	5	3	6	2	7	2
RIPK1	176	396	212	112	123	350	120	129	3720	3743	2798	2330	2684	2843
EVA1B	20	56	94	102	352	62	155	33	3	1	0	12	0	0
IGSF3	335	317	46	135	153	133	776	195	0	2	0	3	2	0
LCN2	0	0	0	0	0	0	1	0	168	13	238	171	817	66
TCF7L1	37	10	48	135	56	24	47	25	1	0	1	2	3	1
VSIG4	96	20	39	72	30	61	30	121	0	1	0	3	0	0
PDZD9	15	9	39	23	10	13	18	11	0	0	0	0	0	0
C1QC	43	5	7	158	7	43	70	80	0	0	0	0	1	1
CHRNB2	186	57	350	165	94	123	138	171	2	2	0	14	0	2
GRIN2C	28	20	29	126	15	30	105	40	0	0	2	4	0	1
VCAM1	99	138	409	1757	139	81	1009	138	0	0	0	0	0	0
SPRR3	0	0	0	0	0	0	0	0	925	149	1015	971	4105	372
SPRR2D	1	0	0	1	0	0	0	0	160	19	38	51	411	69
CTLA4	159	100	168	854	93	59	672	298	5	12	3	6	1	8
RNASE7	1	0	0	1	1	0	0	1	20	4	33	94	174	31
C15orf48	2	1	1	16	0	0	3	0	100	22	117	22	438	51
CXCL8	0	2	0	1	4	0	8	5	30	33	89	51	217	147
CRCT1	0	0	0	0	0	0	0	0	25	2	7	37	19	3
FOS	490	236	113	2534	321	260	1225	369	19,027	13,902	26,445	6251	17,983	39,313
KRT78	1	0	1	0	0	0	1	0	88	10	86	392	295	35
GIMAP8	131	35	56	655	39	19	459	79	1	1	0	2	2	0
KRT13	1	1	0	0	0	0	0	1	256	16	378	671	918	65
ADCY5	46	23	40	413	28	21	249	27	2	1	3	3	1	0
C1QA	119	4	12	250	19	89	99	134	0	0	0	1	0	0
ODF3	29	17	30	127	11	16	29	34	0	0	0	1	0	0
TMPRSS9	52	1	30	175	2	36	19	8	0	0	0	0	1	0
SRPK3	69	28	60	205	27	24	88	31	0	0	0	5	0	1
TMPRSS11B	0	0	1	0	1	0	0	1	160	21	277	440	759	60
ZACN	1029	140	1061	984	595	593	1025	773	6	10	9	62	7	4
SIGLEC15	30	17	53	61	24	27	102	18	0	1	0	2	0	1
LYPD2	0	0	0	3	1	0	0	1	14	3	22	33	75	0
RNA5S1	21	8	2	6	10	50	2	6	399	344	873	51	492	745
SPDYE2	80	83	29	330	36	20	295	63	1	5	0	8	0	0
SMTNL1	47	11	33	75	20	0	50	0	0	0	0	0	0	0
CERS1	7	10	9	32	15	13	22	10	0	0	0	2	0	0
CACTIN-AS1	37	8	16	66	17	14	52	19	0	0	0	2	0	0
EHBP1-AS1	22	15	17	35	15	22	23	18	0	1	0	2	0	0
SNORD13	2	1	2	7	0	1	2	6	101	37	43	4	58	18
SPRR2A	0	0	0	0	0	0	0	0	227	58	64	117	666	133
LINC00861	406	79	113	1910	79	57	322	368	2	1	8	8	1	4
RPL36A-HNRNPH2	13	0	12	0	3	15	17	13	240	255	328	3	246	371
NDUFC2-KCTD14	34	12	13	12	26	25	22	35	544	450	789	0	360	504
TEX52	21	24	58	117	34	39	85	19	0	1	0	5	1	0

### Cell proliferation, viability assay

Cell proliferation was determined using Cell Count Kit-8 (CCK-8, Cat: 40203ES60, YEASEN, Shanghai, China). Cell viability was determined using CellQuanti-Blue^™^ Cell Viability Assay Kit (CQBL-05 K, BioAssay systems). About 5000 cells (including RIPK1-knockdown cells GM12878/L428 and RIPK1 overexpression cells TMD8/U2932) were seeded in a 96-well microplate containing a culture growth medium for 12 h. Then, cells were subjected to a cell proliferation/viability assay every 12 h (0, 12, 24, 36, and 48 h) following the manufacturer’s instruction. Each sample was detected with at least three replications.

### Cell cycle analysis

Propidium Iodide (P1304MP, ThermoFisher Scientific, USA) staining was employed to determine cell cycles. In detail, the collected cells were aliquoted to 1 × 10^6^ cells/100 μL into FACS tubes, followed by twice washing using 1xPBS. Then, cells were centrifuged for 5 min at a speed of 300×g. Furthermore, cells were resuspended using 100 μL of Flow Cytometry Staining Buffer (Cat: # FC001, R&D systems). 10 μL of PI staining solution was added to a control tube of otherwise unstained cells to adjust flow cytometer settings for PI. Cells were mixed gently and incubated for 5 min in the dark. PI fluorescence of samples was determined (using the FL-2 or FL-3 channel) with a FACScan™ instrument. Each detection was repeated at least 3 times. For cell death ratio detection, Annexin V-FITC/PI double-labeled FACS was carried out following the manufacturer’s protocol (Cat: 40302ES20, Yeasen, Shanghai). Flowjo software was used for gate setting FACS original data, and the ggcyto R package (https://bioconductor.org/packages/release/bioc/html/ ggcyto.html) was used for visualization.

### Public data analysis

The public high throughput sequencing data were downloaded from the Gene Expression Omnibus database, which includes CD19^+^ B-cells from primary peripheral blood or lymph node biopsies of normal participants (N = 6), chronic lymphocytic leukemia (CLL, N = 5), follicular lymphoma (FL, N = 10), and diffuse large b cell patients (DLBCL, N = 8).

(GSE145842, https://www.ncbi.nlm.nih.gov/geo/query/acc.cgi?acc=GSE145842).

The analysis of differentially expressed genes (DEGs) was performed using an R package of DEseq2 (version-1.36, https://bioconductor.org/packages/release/bioc/html/DESeq2.html), with a cutoff of p-adjust < 0.001 and log2 fold changes > 3. The DEGs identified were visualized by volcano plot using an R package of EnhancedVolcano (version-1.14, https://bioconductor.org/packages/release/bioc/html/EnhancedVolcano.html).

### Statistical analysis

In this study, data were presented as mean ± (SD), and the replications of all the samples were presented in the figure legends. The software of GraphPad Prism9 (San Diego, USA) was employed for statistical analyses. The student’s *t*-test was utilized for comparisons between two groups, and a one- or two-way ANOVA test for comparisons between three or more groups. A *P* value < 0.05 was set as the cutoff of statistical significance.

## Results

### RIPK1 is down-regulated in tumor cells of lymphoma patients

**Fig. 1:**
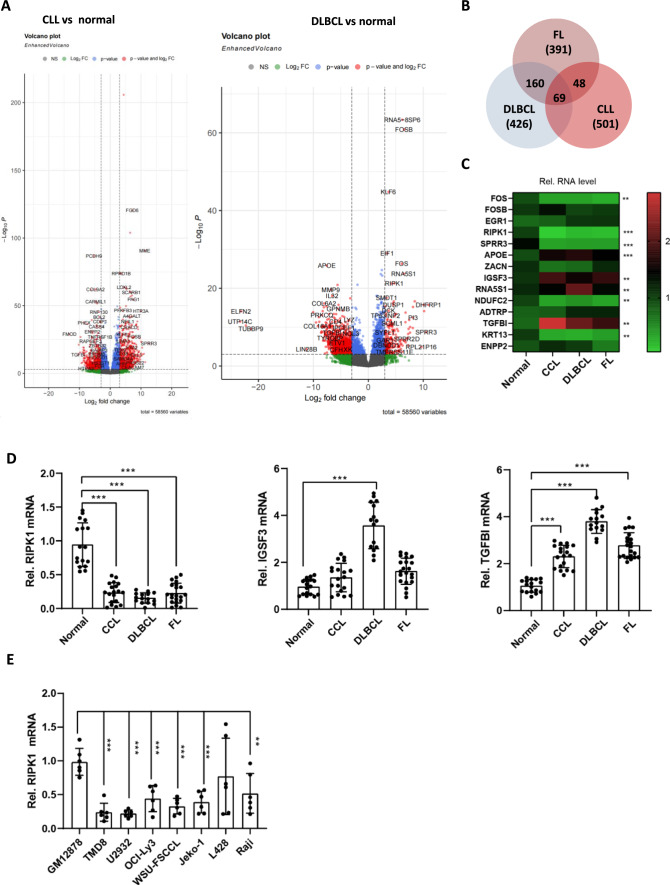
RIPK1 is down-regulated in tumor cells of lymphoma patients. **A** Volcano plot of DEGs of CLL and DLBCL vs. normal donors, respectively. Chronic lymphocytic leukemia (CCL, N = 5) and diffuse large B-cell patients (DLBCL, N = 8) vs. normal donors (N = 6). The cutoff was set at p-adjust < 0.001 and log2 fold change > 3. **B** Venn gram of three groups of DEGs. CLL and DLBCL vs. normal donor from 1A, FL vs. normal donor from S1B, all the genes in detail were listed in Table 3. **C** Real-time PCR estimated the mRNA level of the top 14 highly expressed DEGs in B. The CD19^+^ B-cells of 17 normal donors, 19 CCL, 15 DLBCL, and 21 FL were pooled together, respectively, and then were subjected to real-time PCR. The color shows the mean of the relative expression of each group. N = 3. **P < 0.01 and ***P < 0.001 by one-way ANOVA. **D** Real-time PCR estimated the mRNA level of 3 DEGs in CD19^+^ B-cells from the indicated participants. Samples are the same with C. **P < 0.01 and ***P < 0.001 by student’s *t*-test.** E** Real-time PCR estimated the mRNA level of RIPK1 in 8 lines of B-cells. Normal human B lymphocyte cell line (GM12878), B-cell non-Hodgkin lymphoma (OCI-LY3, JEKO-1, RAJI, TMD8, U2932, WSU-FSCCL), and a Hodgkin's lymphoma cell line (L428). N = 6, ***P < 0.001 by student’s *t*-test

To identify a common target for B-cell lymphoma in treatment, we analyzed a CD19^+^ B-cell transcriptomic dataset from the Gene Expression Omnibus (GEO) database, which consists of samples from normal participants (N = 6), chronic lymphocytic leukemia (CLL, N = 5), follicular lymphoma (FL, N = 10), and diffuse large B-cell lymphoma patients (DLBCL, N = 8). Firstly, we identified three groups of differentially expressed genes (DEGs) by comparing CLL, DLBCL, and FL with normal controls, respectively, and then calculated the common DEGs (Fig. 1A and Additional file 1: S1A, B). Fortunately, we obtained 69 common DEGs (Fig. 1B, Table 3). Then, we selected the top 14 high-expressed DEGs and validated the expression in our B-cell lymphoma samples (19 CLL, 21 FL, and 15 DLBCL) using real-time PCR (Fig. 1C). In the pooled samples' real-time PCR, 64.2% (9/14) DEGs were successfully confirmed, such as FOS, RIPK1, SPRR3, APOE, etc. (Fig. 1C). Furthermore, we detected the RNA level of these 9 DEGs in individual samples mentioned above, three of which were further validated, including RIPK1, IGSF3, and TGFB1 (Fig. 1D). In detail, the expression level of RIPK1 was dramatically down-regulated both in the CD19^+^ B-cells of CLL, DLBCL, and FL when compared with that in normal donors, and in contrast, IGSF3 and TGFB1 expression levels were up-regulated (Fig. 1D). Besides, we estimated the expression of these three genes in 8 lymphocyte lines, including a normal human B lymphocyte cell line (GM12878), and 6 B-cell non-Hodgkin lymphoma (OCI-LY3, JEKO-1, RAJI, TMD8, U2932, WSU-FSCCL) and a Hodgkin's lymphoma cell line (L428). Interestingly, we observed that RIPK1 mRNA levels were decreased in B-cell lymphoma cell lines when compared with GM12878 normal B-cells (Fig. 1E). Taken together, our results indicated that RIPK1 is commonly down-regulated in B-cell lymphoma cells.

**Table 3:** Oligos used in this study for real-time PCR

Oligos	Forward(5ʹ —3ʹ)	Reverse (5ʹ—3ʹ)
FOS	GGGGCAAGGTGGAACAGTTAT	CCGCTTGGAGTGTATCAGTCA
FOSB	GCTGCAAGATCCCCTACGAAG	ACGAAGAAGTGTACGAAGGGTT
EGR1	CCACGCCGAACACTGACATT	GAGGGGTTAGCGAAGGCTG
RIPK1	TGGGCGTCATCATAGAGGAAG	CGCCTTTTCCATGTAAGTAGCA
SPRR3	ATGAGTTCTTACCAGCAGAAGC	GTTCAGGGACCTTGGTGTAGC
APOE	GTTGCTGGTCACATTCCTGG	GCAGGTAATCCCAAAAGCGAC
ZACN	CCTGGGGTTCAGCATTACCTT	GTTGACGTTGAAGAGGGATGG
IGSF3	GCCGAGATTTCATGCTTCACT	CGGAACGCTTTCGGGTCATA
RNA5S1	AACGCGCCCGATCTCGTC	AGGCGGTCTCCCATCCAAGTA
NDUFC2	ACCCAGAACCCTTACGGTTTC	CTCCGCCGGATCAGGTTATC
ADTRP	ACCCCAAGGTCCTAGATACTGT	AAAGCTGCTAGACCCAAGAGG
TGFBI	CTTCGCCCCTAGCAACGAG	TGAGGGTCATGCCGTGTTTC
KRT13	GACCGCCACCATTGAAAACAA	TCCAGGTCAGTCTTAGACAGAG
ENPP2	TCGCTGTGACAACTTGTGTAAG	CCAATGCGACTCTCCTTTGC
GAPDH	CTGGGCTACACTGAGCACC	AAGTGGTCGTTGAGGGCAATG

### Loss-function of RIPK1 contributes to the cell growth of lymphocytes

**Fig. 2:**
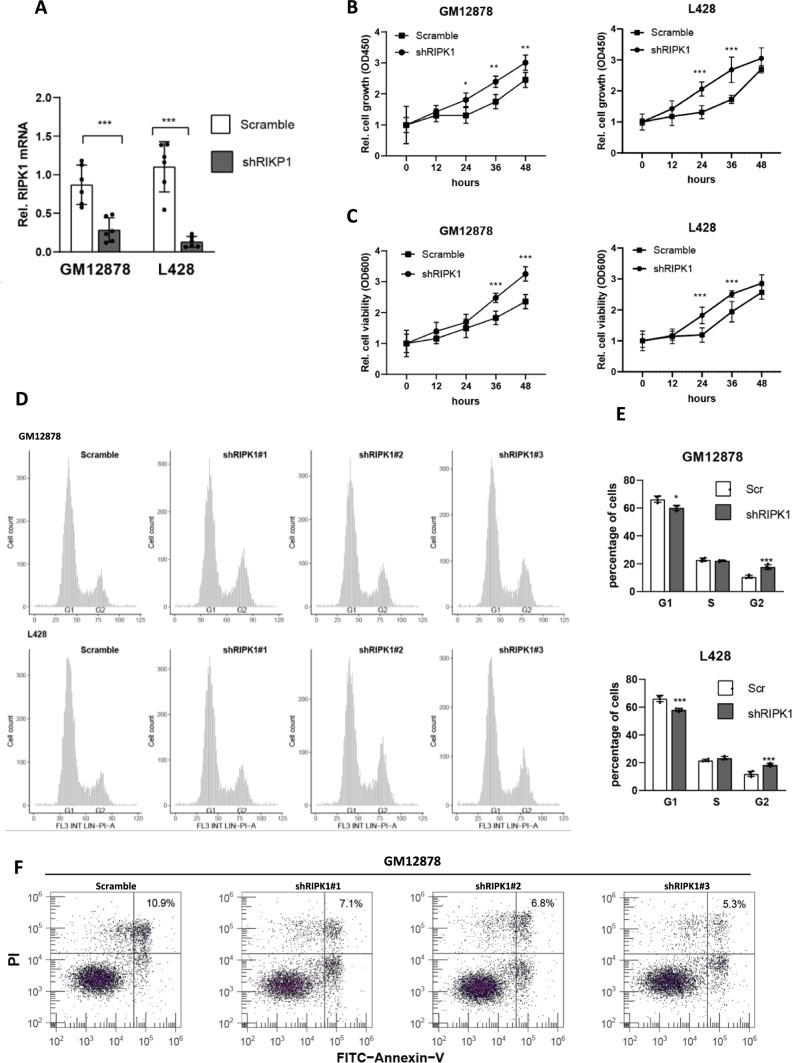
Loss-function of RIPK1 contributes to the cell growth of lymphocytes **A** Real-time PCR estimated the RIPK1 knockdown efficiency in GM12878 and L428 cells. Scramble, scramble shRNA without a specific target; shRIPK1, a pool of three independent shRNAs targeting RIPK1. N = 6, ***P < 0.001 by student’s t-test. **B** CCK8 assay determined the cell growth of GM12878 and L428 cell lines. Cells in A were seeded onto 96-well plates, and 12 h later, cell growth status was detected every 12 h. N = 3, *P < 0.05, **P < 0.01, ***P < 0.001 by two-way ANOVA. **C** Cell viability of GM12878 and L428 cell lines. Cells in A were seeded onto 96-well plates, and 12 h later, cell viability was detected every 12 h. N = 3, *P < 0.05, **P < 0.01 by two-way ANOVA. **D**–**E**. PI staining determined the cell cycle distribution of GM12878 and L428 cell lines. ~ 5000 cells cultured for 36 h were collected for PI staining. shRIPK1#1/2/3 indicates independent shRNA targets of RIPK1. Scr, scramble shRNA; N = 6, ***P < 0.001 by student’s *t*-test. **E** Annexin V-FITC/PI double-labeled FACS determined the cell death of GM12878 cell lines. Cells in D were used for experiments

The aberration of RIPK1 has been extensively established to be implicated in the pathogenesis of multiple categories of solid cancers by altering cell survival, such as pancreatic, colorectal, hepatocellular cancers, etc. [18–20]. Therefore, we speculated that the down-regulation of RIPK1 may also play a pathogenic role in B-cell lymphomas. Then, we knock down the expression of RIPK1 in GM12878 (a normal human B lymphocyte cell line) and L428 (a Hodgkin's lymphoma cell line with a relatively high expression of RIPK1) cells using the shRNA vector (Fig. 2A). In the CCK8 assay, we observed that knockdown of RIPK1 in GM12878 cells significantly promoted cell proliferation after 24 h of seeding, and consistently, L428 cells of the shRIPK1 group also dramatically faster than that of the scramble group (Fig. 2B). These results indicate that RIPK1 possesses an inhibitory function in the cell proliferation of B lymphocytes. In the cell viability assay, our results showed that the cell viability of the shRIPK1 group (both in GM12878 and L428 cells) was elevated after 24 h of seeding when compared with that of the scramble group (Fig. 2C). Furthermore, the cell cycle was analyzed using propidium iodide (PI) staining assay. We observed that the loss function of RIPK1 using shRNA in GM12878 cells significantly increased the G2 stage percentage, from 14.3% (in ~ 5000 cells) in the scramble group to 19.8% in the shRNA group (Fig. 2D, E). Consistently, similar results were also found when comparing the shRIPK1 L428 with the scramble one (Fig. 2D, E). These results indicated that RIPK1 possesses an inhibitory function in the proliferative ability of B lymphocytes. Besides, we observed that the loss-function of RIPK1 using shRNA inhibited cell death, evidenced by a reduction of 37.6% and 35.9% of cell death in GM12878 and L428 cells, respectively (Fig. 2F and Additional file 1: S2E). Altogether, the loss function of RIPK1 contributes to the cell growth of lymphocytes.

### Ectopic RIPK1 in lymphocytes suppresses cell growth

**Fig. 3:**
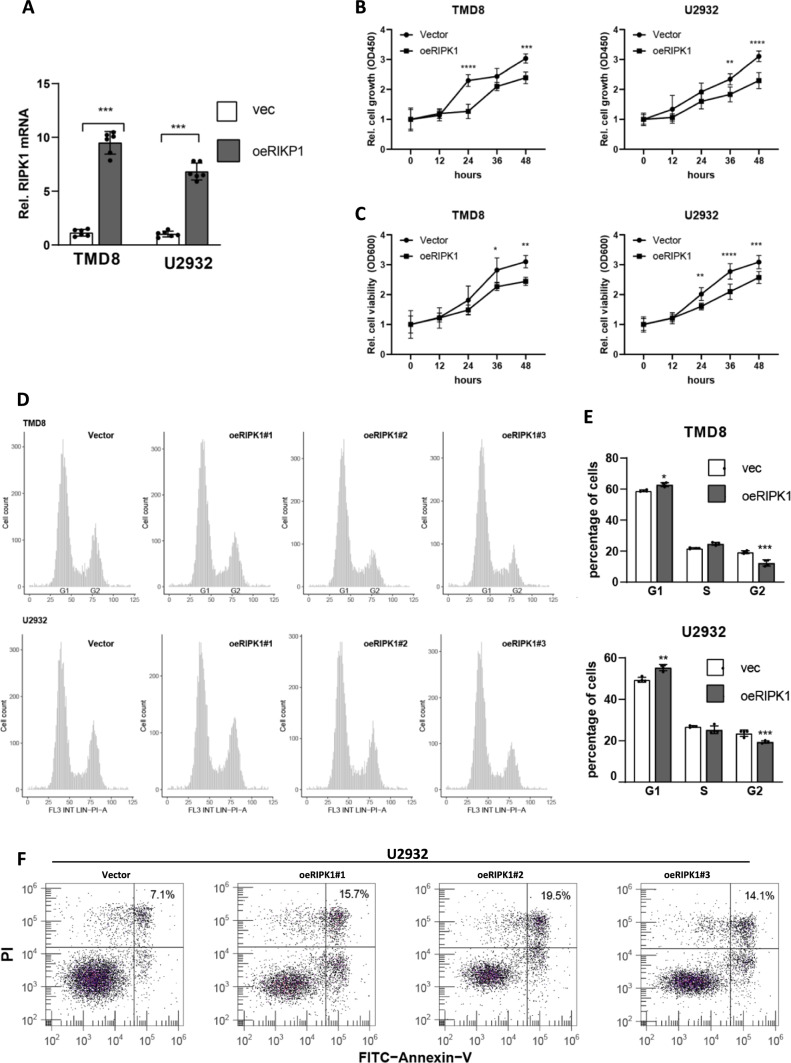
Ectopic RIPK1 in lymphocytes suppresses cell growth **A** Real-time PCR estimated the RIPK1 knockdown efficiency in TMD8 and U2932 cells. N = 6, ***P < 0.001 by student’s *t*-test. **B** CCK8 assay determined cell proliferation of TMD8 and U2932 cell lines. Cells in A were seeded onto 96-well plates, and 12 h later, cell growth status was detected every 12 h. Vector, empty vector; oeRIPK1, RIPK1 overexpression. N = 3, *P < 0.05, **P < 0.01, ***P < 0.001 by two-way ANOVA. **C** Cell viability of TMD8 and U2932 cell lines. Cells in A were seeded onto 96-well plates, and 12 h later, cell growth status was detected every 12 h. Vector, empty vector; oeRIPK1, RIPK1 overexpression. N = 3, **P < 0.01, ***P < 0.001 by two-way ANOVA. **D**–**E**. PI staining determined the cell cycle distribution of TMD8 and U2932 cell lines. ~ 5000 cells cultured for 36 h were collected for PI staining. oeRIPK1#1/2/3 indicates three duplications of RIPK1 overexpressed cell lines. Vector, empty vector; oeRIPK1, RIPK1 overexpression. N = 6, *P < 0.05, **P < 0.01, ***P < 0.001 by student’s *t*-test **F**. Annexin V-FITC/PI double-labeled FACS determined the cell death of U2932 cell lines. Cells in B were used for experiments.

To further validate RIPK1’s inhibitory function in B-cell proliferation, we utilized a lentivirus vector to construct RIPK1 stably overexpressed cell lines in TMD8 and U2932 cells, two RIPK1 low-expressed DLBCL cell lines (Fig. 1E). After quantification of RIPK1 using real-time PCR, we found RIPK1 expression was elevated to 9.5-fold and 6.8-fold in TMD8 and U2932, respectively (Fig. 3A). In the CCK8 assay, our results showed that, after 24 h of seeding, the cell growth of RIPK1-overexpressed TMD8 cells was significantly slower than that of the empty vector group (Fig. 3B). Consistently, similar results also were observed in RIPK1-overexpressed U2932 cells (Fig. 3B). These results directly reveal that RIPK1 possesses an inhibitory function in the cell proliferation of B lymphocytes. Meanwhile, we evaluated the cell viability assay for RIPK1-overexpressed TMD8 and U2932 cell lines (Fig. 3C). Our results indicated that the cell viability of RIPK1-overexpressed U2932 cells was decreased after 24 h of seeding, which is similar to that of TMD8 cells (since 36 h) (Fig. 3C). Furthermore, we carried out cell cycle analysis for RIPK1-overexpressed TMD8 and U2932 cell lines using PI staining assay. We observed that ectopic RIPK1 in TMD8 cells significantly decreased the G2 stage percentage, from 19.3% (in ~ 5000 cells) in the empty vector group to 13.4% in the RIPK1 group (Fig. 3D, E). Similarly, we also observed G2 stage percentage decreased from 23.7% to 19.4% in RIPK1 overexpressed U2932 cells. In addition, the cell death ratio was increased by 1.1 and 0.8 folds after overexpression of RIPK1 in U2932 and TMD8 cells, respectively (Fig. 3F and Additional file 1: S3E). Altogether, the loss function of RIPK1 contributes to the cell growth of lymphocytes. Therefore, our findings collectively revealed that RIPK1 possesses inhibitory functions in the proliferative ability of B lymphocytes.

### RIPK1 inhibitor promotes the proliferation of lymphoma cells

**Fig. 4:**
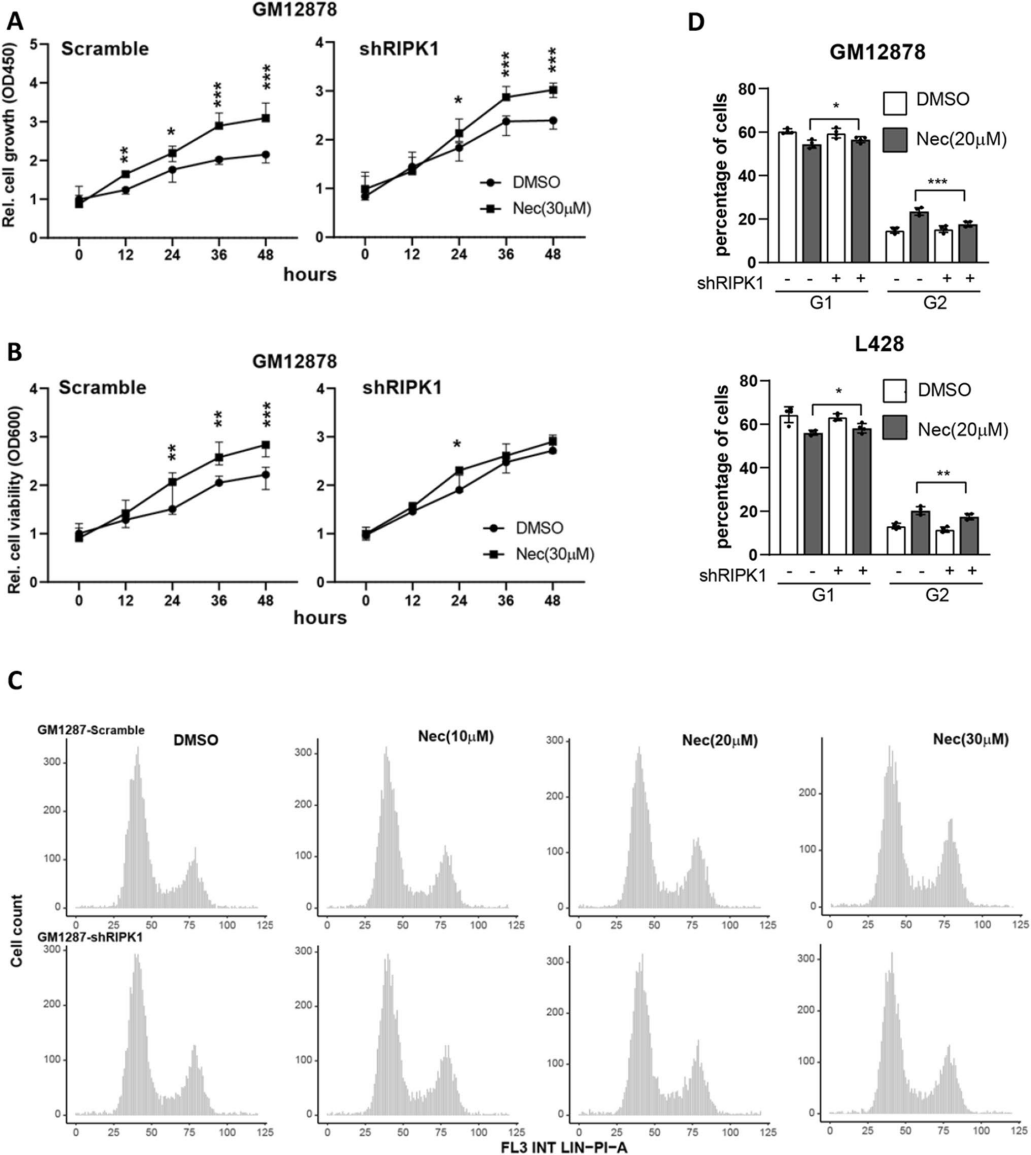
RIPK1 inhibitor promotes the proliferation of lymphoma cells **A** CCK8 assay determined cell proliferation of GM12878 lines. Cells were seeded onto 96-well plates and pre-treated with Nec (30 μM) for 24 h, and then, cell growth status was detected by CCK8 assay every 12 h. N = 3, *P < 0.05, **P < 0.01, ***P < 0.001 by two-way ANOVA. **B** Cell viability of GM12878 cell lines. Cells in A were seeded onto 96-well plates and pre-treated with Nec (30 μM) for 24 h, and then, cell growth status was detected every 12 h. N = 3, *P < 0.05, **P < 0.01, ***P < 0.001 by two-way ANOVA. C-D. PI staining **C** determined the cell cycle distribution of GM12878 cell lines and the statistical results **D**. ~ 5000 cells were pre-treated with Nec (30 μM) for 24 h and then collected for PI staining. N = 6, *P < 0.05, ***P < 0.001 by student’s *t*-test

A bulk of studies have indicated that RIPK1 has a dual biological role: 1) promoting cell apoptosis and necroptosis in a kinase activity-dependent manner, and 2) promoting cell survival via its scaffolding function in TNFR1 (tumor necrosis factor receptor 1) signaling [21, 22]. Necrostatin-1 (Nec), as the first specific inhibitor of RIPK1, has been extensively demonstrated to possess noticeable effects on inhibiting necroptosis in various diseases, including inflammatory cardiovascular, neurological diseases, etc. [23]. Therefore, we explored the effects of Nec on B-cell lymphoma. In the cell proliferation assay, we observed that Nec treatment (30 μM, based on the previous report [24]) on GM12878/L428 cells significantly promoted cell growth, which was partially blocked by downregulated RIPK1 expression using shRNA (Fig. 4A and Additional file 1: S2A). Meanwhile, cell viability also was increased by Nec treatment on GM12878/L428 cells, and these effects were largely attenuated in the RIPK1 knockdown group (Fig. 4B and Additional file 1: S2B). Furthermore, we observed that Nec treatment elevated G2 percentage both on GM12878 and L428 cells in a dose-dependent manner, and effects were partially abated in RIPK1 knockdown groups (Fig. 4C–D and Additional file 1: S2C). These results indicate that Nec promotes cell proliferation of B-cell lymphoma largely via targeting RIPK1. Besides, we treated GM12878 and L428 cells using another RIPK1 inhibitor, RIPK1-IN-7 (IN-7), which has a low enzymatic IC50 of 11 nM [25]. In our experiments, we found that treatment of IN-7 (5 nM) increased G2 percentage both in GM12878 and L428 cells, which were largely attenuated in RIPK1 knockdown cells (Additional file 1: Fig. S2D). Altogether, inhibition of RIPK1 kinase activity by small molecule inhibitors can efficiently promote cell proliferation.

### HOIPIN-1 inhibits the proliferation of lymphoma cells by enhancing RIPK1 function

**Fig. 5:**
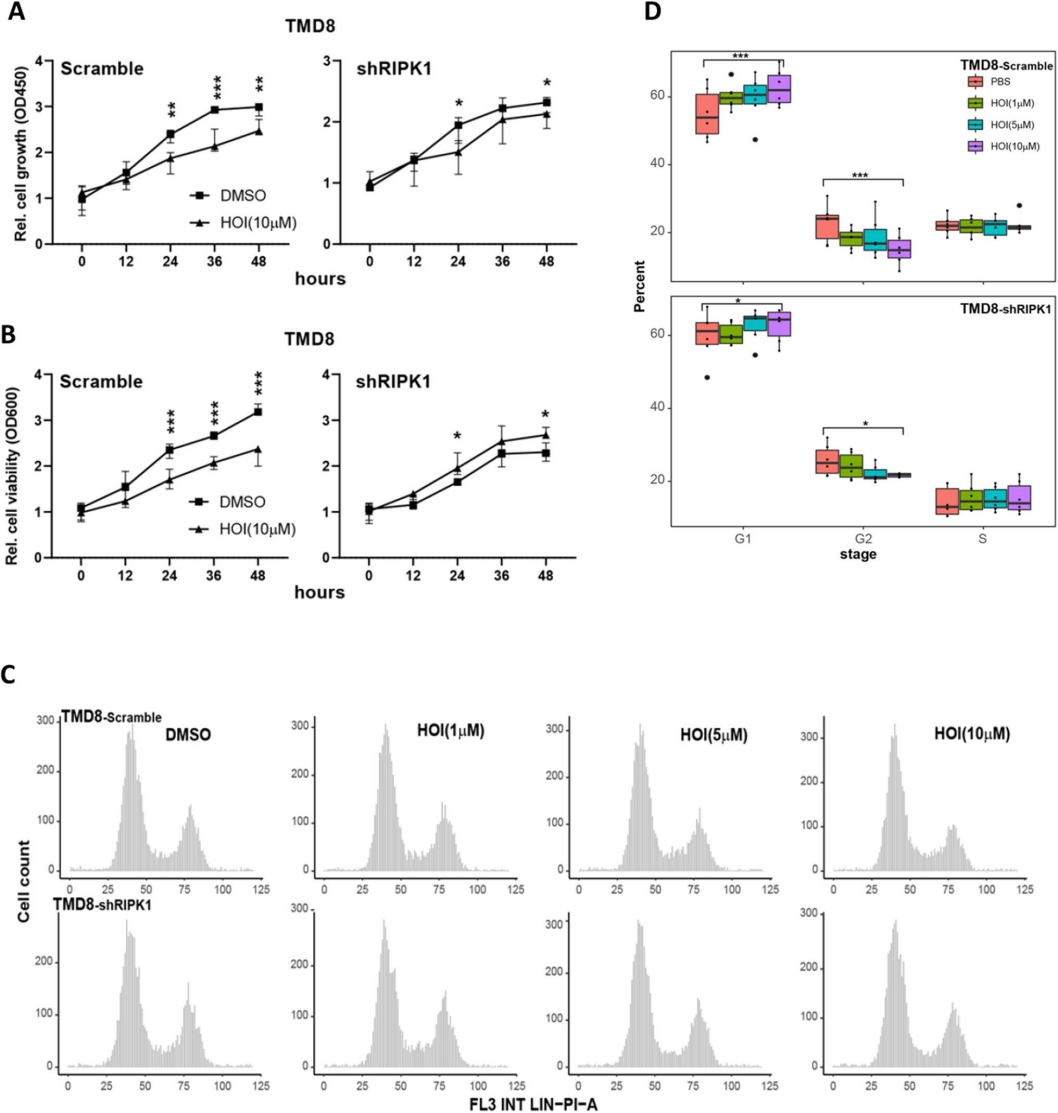
HOIPIN-1 inhibits the proliferation of lymphoma cells by blocking RIPK1 function **A**. CCK8 assay determined cell proliferation of TMD8 lines. Cells were seeded onto 96-well plates and pre-treated with HOIPIN-1 (HOI, 10 μM) for 24 h, and then, cell growth status was detected by CCK8 assay every 12 h. N = 3, *P < 0.05, **P < 0.01, ***P < 0.001 by two-way ANOVA. **B** Cell viability of TMD8 cell lines. Cells in A were seeded onto 96-well plates and pre-treated with HOI (10 μM) for 24 h, and then, cell viability status was detected every 12 h. N = 3, *P < 0.05, **P < 0.01, ***P < 0.001 by two-way ANOVA. **C**–**D**. PI staining (C) determined the cell cycle distribution of TMD8 cell lines and the statistical results (D). ~ 5000 cells were pre-treated with HOI at the indicated dose for 24 h and then collected for PI staining. N = 6, *P < 0.05, ***P < 0.001 one-way ANOVA

Due to the down-regulation of RIPK1 expression of kinase activity promoting cell proliferation, we speculated that activation of RIPK1 by small molecules may lead to cell death of B-cell lymphoma. However, to date, there is still no effective and specific RIPK1 agonist available to activate this enzyme in experiments. Numerous previous studies showed that there is a balance between the kinase activity and scaffold function of RIPK1 [9, 26, 27]. Moreover, modulation of RIPK1 scaffold functions via ubiquitination-related enzymes (such as IAP, LUBAC, A20, and CYLD) can largely influence RIPK1-mediated signaling [28]. Therefore, we hypothesized that LUBAC inhibitors, such as HOIPIN-1 (with an IC50 of 2.8 μM [29]) may enhance RIPK1 kinase-mediated cell death by abating its ubiquitination. To this end, we treated TMD8 and U2932 cells with HOIPIN-1 (HOI) and checked the growth status of the cells. Indeed, we observed that HOI treatment (10 μM) on TMD8 and U2932 cells dramatically suppressed cell growth, and these effects were largely attenuated by the RIPK1-knockdown group (Fig. 5A and Additional file 1: S3A). Consistently, cell viability also decreased after HOI treatment on GM12878/L428 cells, which were abated by the knockdown of RIPK1 expression (Fig. 5B and Additional file 1: S3B). In the cell cycle analysis, we observed that HOI treatment decreased G2 percentage both in TMD8 and U2932 cells in a dose-dependent manner (Fig. 5C–D and Additional file 1: S3C–D). More importantly, these effects were effectively attenuated after the knockdown of RIPK1 using shRNA (Fig. 5C–D and Additional file 1: S3C–D). These results indicate that 1) HOI treatment inhibits cell proliferation of B-cell lymphoma largely via targeting RIPK1; 2) restoration of RIPK1 by small molecule inhibitors can efficiently promote cell death of B-cell lymphoma.

## Discussion and conclusion

A bulk of reports have demonstrated that RIPK1 generally functions as a critical scaffold molecule of the TNFR1 complex, and achieves various inflammatory and pro-survival biological activities by modulating the activation of the MAPK and NF-κB signaling pathways [10, 22]. Meanwhile, as a kinase, RIPK1 physically interacts with RIPK3 to paradoxically promote apoptosis, necroptosis, etc. by catalyzing the phosphorylation of MLKL [30–32]. The dual role of RIPK1 in cell survival emphasizes that the unbalance of RIPK1 expression or activity may cause a serious outcome for cells. In our study, we observed that the mRNA level of RIPK1 was significantly down-regulated in the main categories of B-cell lymphoma, including CLL, DLBCL, and FL by re-analyzing the public dataset in the GEO database. Meanwhile, we also validated these results using biopsies we collected by real-time quantitative PCR. These results suggest that characterizing the roles of RIPK1 in B-cell lymphoma may identify a common target for potential therapy of these diseases.

Firstly, we estimated the expression of RIPK1 in 8 lymphocyte lines, including a normal human B lymphocyte cell line (GM12878), and 6 B-cell non-Hodgkin lymphomas (OCI-LY3, JEKO-1, RAJI, TMD8, U2932, WSU-FSCCL) and a Hodgkin's lymphoma cell line (L428). Indeed, we observed that RIPK1 mRNA levels were decreased in 6 B-cell lymphoma cell lines when compared with normal B-cell GM12878. However, RIPK1 in L428 is slightly (but not significantly) lower than that of GM12878 cells. These findings suggest that the down-regulation of RIPK1 may be common in B-cell but not all subtypes of lymphoma cells. However, this conclusion needs more evidence to support. In 2021, Yin et al. reported that RIPK1 depletion occurs in triple-negative breast carcinoma, and the loss function of RIPK1 significantly aggravates the AQP1-driven TNBC progression and metastasis [33]. More recently, Yu et al. demonstrated that SMYD2 contributes to colorectal cancer by targeting RIPK1 and inhibiting the phosphorylation of RIPK1 [34]. Combined with our observations, it is obvious that the aberration of RIPK1 kinase activity contributes to the pathogenesis of multiple cancers, both solid and blood cancers.

To further investigate the roles of RIPK1 in B-cell lymphoma, we knock down the expression of RIPK1 in GM12878 and L428 cells. Interestingly, our results indicate that the knockdown of RIPK1 significantly promotes cell growth and viability, and inhibits cell death both in GM12878 and L428 cells. Meanwhile, we also observed that the G2 percentage is elevated after RIPK1 down-regulation. In contrast, overexpression of RIPK1 in TMD8 and U2932 cells inhibits cell growth and viability and decreases G2 percentage, and promotes cell death. These results support the previously established kinase-dependent pro-apoptosis and -necroptosis functions of RIPK1. Furthermore, we employed two different RIPK1 inhibitors, Necrostatin-1 and RIPK1-IN-7, to confirm that the kinase activity of RIPK1 plays a critical role in the cell growth of normal B lymphocytes and other types of lymphoma cell lines. Our data reveal that inhibition of RIPK1 kinase activity contributes to cell growth of normal B-cells, which is in favor of the RIPK1 knockdown results mentioned above. Noticeably, necrostatin-1, the inhibitor of necroptosis, has been explored to be developed into a small molecule drug for inflammation-, apoptosis- or necroptosis-related diseases, such as cardiovascular diseases, neonatal hypoxia–ischemia, Alzheimer’s disease, Parkinson’s disease, coronavirus disease 2019 (COVID-19), etc. [23, 35–37]. According to our evidence, necrostatin-1 (or RIPK1 inhibitors) may increase the risk of B-cell lymphoma occurrence.

On the other hand, we speculated that the RIPK1 agonist may exhibit an effect against the cell growth of B-cell lymphoma. However, no effective and specific RIPK1 agonist is available to date. It has been well established that the ubiquitination of RIPK1 is mainly mediated by E3 ubiquitin ligases LUBAC, by which the kinase activity of RIPK1 is indirectly arrested [38]. To this end, we used HOI to treat TMD8 and U2932 cells. Interestingly, we observed that HOI treatment dramatically suppressed cell growth of TMD8 and U2932, and increased the G1 arrest of these two cell lines. These findings reveal that restoration of RIPK1 by small molecule inhibitors can efficiently promote cell death of B-cell lymphoma. Notably, HOI is well-known as an inhibitor of the canonical NF-kB by suppressing LUBAC ubiquitin ligase activity [39]. In our recent results (data not shown), we also observed that the p65 phosphorylation (the activated NF-kB subunit) level was significantly decreased by HOI treatment in both TMD8 and U2932 cells. Meanwhile, we found overexpression of RIPK1 inhibited NF-kB activation, which is similar to HOI treatment. These results suggest that HOI is versatile in suppressing cell apoptosis, via both NF-kB and RIPK1-dependent mechanisms. Besides, considering the dramatic inhibitory effect of RIPK1 specific inhibitor on cell death, we hold that RIPK1 promotes cell death by both kinase- and scaffold-mediated function.

In summary, our study reveals that RIPK1 is down-regulated in B-cell lymphoma, including CLL, DLBCL, and FL. Meanwhile, RIPK1 exhibits anti-tumor activity in a kinase-dependent manner in the context of B-cell lymphoma. More importantly, modifying RIPK1 kinase activity by a small molecule (such as necrostain-1, HOIPIN-1, etc.) alters the cell growth status of B-cell lymphoma. Taken together, we consider that RIPK1 may be a potential target in the clinical application of B-cell lymphoma treatment.

## Supplementary Information

**Additional file 1: Fig S1.** RIPK1 is down-regulated in tumor cells of lymphoma patients. A Heatmap of 3 groups of DEGs (CLL, DLBCL, and FL vs. normal, respectively). Chronic lymphocytic leukemia (CCL, N=5), follicular lymphoma (FL, N=10), and diffuse large B-cell patients (DLBCL, N=8) vs. normal donors (N=6). The cutoff was set at p-adjust < 0.001 and log2 fold change > 3. B Volcano plot of DEGs of FL vs. normal. Follicular lymphoma (FL, N=10) vs. normal donors (N=6). **A** cutoff of p-adjust < 0.001 and log2 fold change > 3. **Fig S2.** RIPK1 inhibitor promotes the proliferation of lymphoma cells A CCK8 assay determined cell proliferation of L428 lines. Cells were seeded onto 96-well plates and pre-treated with Nec (30 μM) for 24 h, and then, cell growth status was detected by CCK8 assay every 12 h. N=3, *P<0.05, **P<0.01, ***P<0.001 by two-way ANOVA. **B** Cell viability of L428 cell lines. Cells in A were seeded onto 96-well plates and pre-treated with Nec (30 μM) for 24 h, and then, cell growth status was detected every 12 h. N=3, *P<0.05, ***P<0.001 by two-way ANOVA. C PI staining determined the cell cycle distribution of L428 cell lines and the statistical results. ~5000 cells were pre-treated with Nec (30 μM) for 24 h and then collected for PI staining. D PI staining determined the cell cycle distribution of GM12878/L428 cell lines and the statistical results. ~5000 cells were pre-treated with RIPK1-IN-7 (IN-7, 5 nM) for 24 h and then collected for PI staining. N=6, ***P<0.001 by student’s *t*-test. E Annexin V-FITC/PI double-labeled FACS determined the cell death of L428 cell lines. Cells in A were used for experiments. **Fig S3.** HOIPIN-1 inhibits the proliferation of lymphoma cells by blocking RIPK1 function. **A** CCK8 assay determined cell proliferation of U2932 lines. Cells were seeded onto 96-well plates and pre-treated with HOIPIN-1 (HOI, 10 μM) for 24 h, and then, cell growth status was detected by CCK8 assay every 12 h. N=3, *P<0.05, **P<0.01, ***P<0.001 by two-way ANOVA. **B** Cell viability of U2932 cell lines. Cells in A were seeded onto 96-well plates and pre-treated with HOI (10 μM) for 24 h, and then, cell growth status was detected every 12 h. N=3, *P<0.05, ***P<0.001 by two-way ANOVA. **C**–**D**. PI staining determined the cell cycle distribution of U2932 cell lines and the statistical results **D**. ~5000 cells were pre-treated with HOI at the indicated dose for 24 h and then collected for PI staining. N=6, *P<0.05, ***P<0.001 by one-way ANOVA.E. Annexin V-FITC/PI double-labeled FACS determined the cell death of TMD8 cell lines. Cells in Fig. 3B were used for experiments.
