# Supplementary material for: RIPK1 is aberrantly expressed in multiple B-cell cancers and implicated in the underlying pathogenesis
Source: Discov Oncol. 2023 Jul 18;14:131. doi: 10.1007/s12672-023-00725-z (PMC10353973; doi:10.1007/s12672-023-00725-z)

Fig.S1 RIPK1 is down-regulated in tumor cells of lymphoma patients

A

CLL vs. normal | DLBCL vs. normal | FL vs. normal

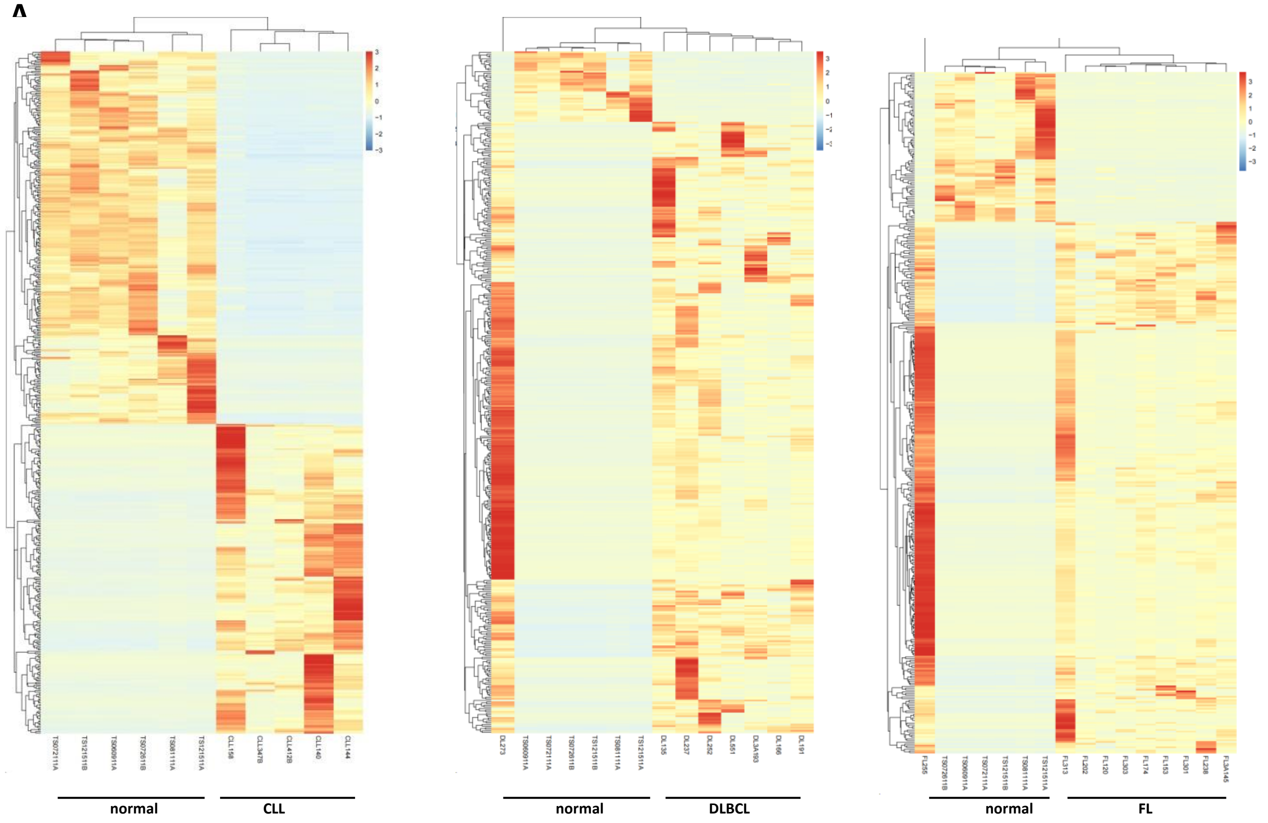

B

FL vs. normal

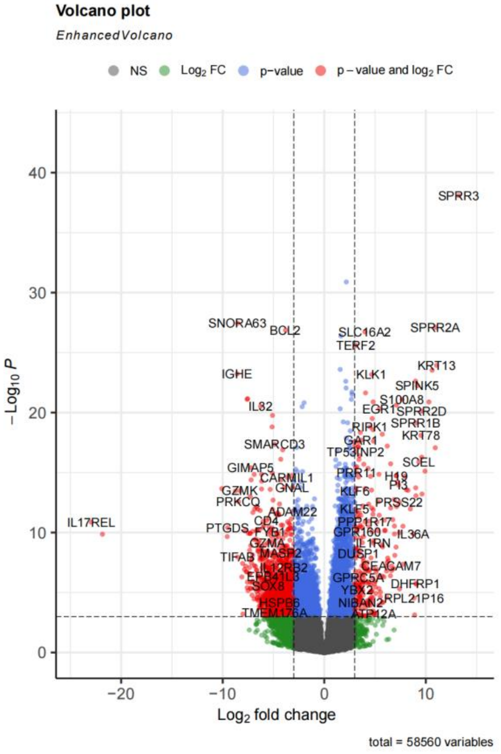

Fig.S2. RIPK1 inhibitor promotes proliferation of lymphoma

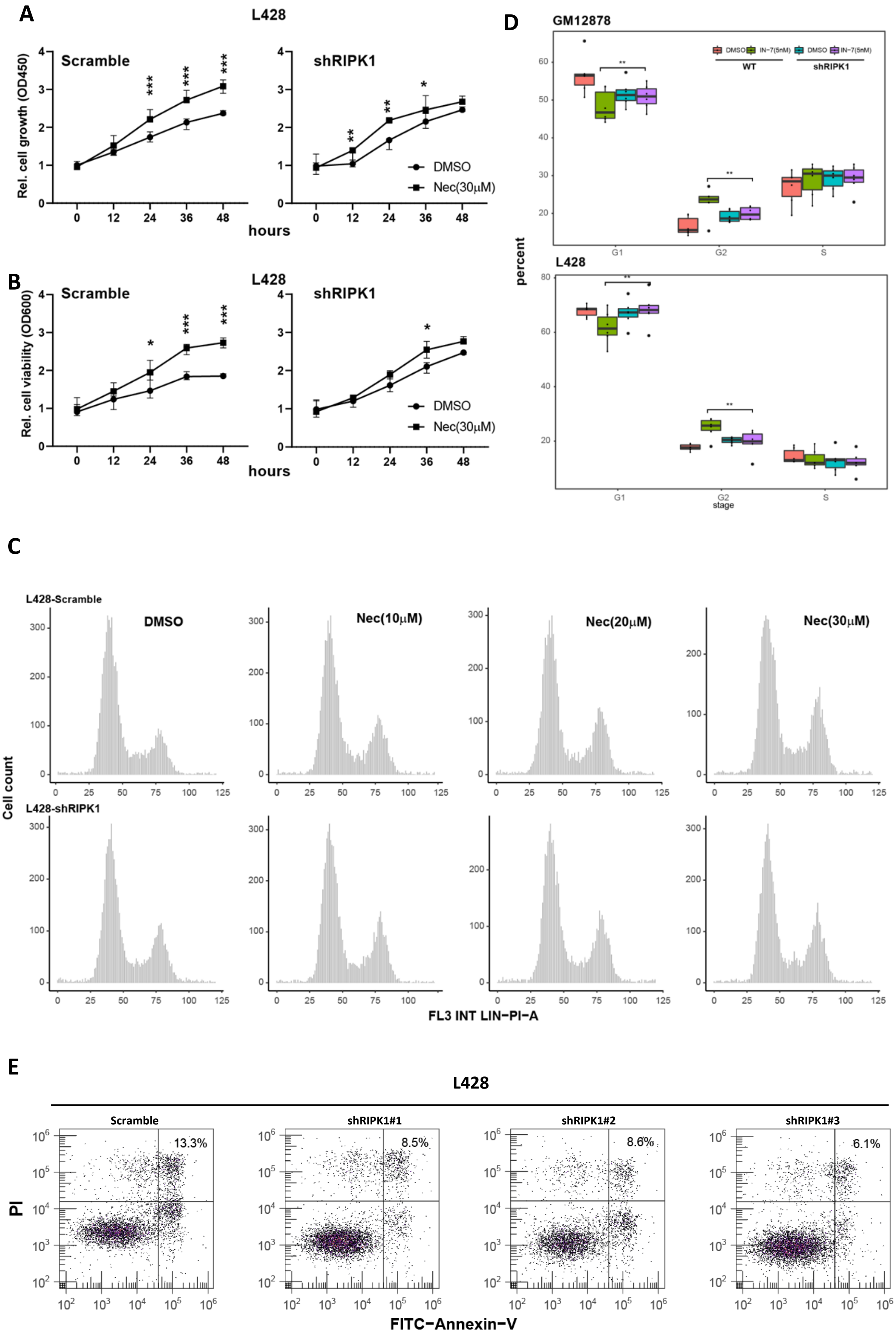

Fig.S3. HOIPIN-1 inhibits proliferation of lymphoma cells by blocking RIPK1 function

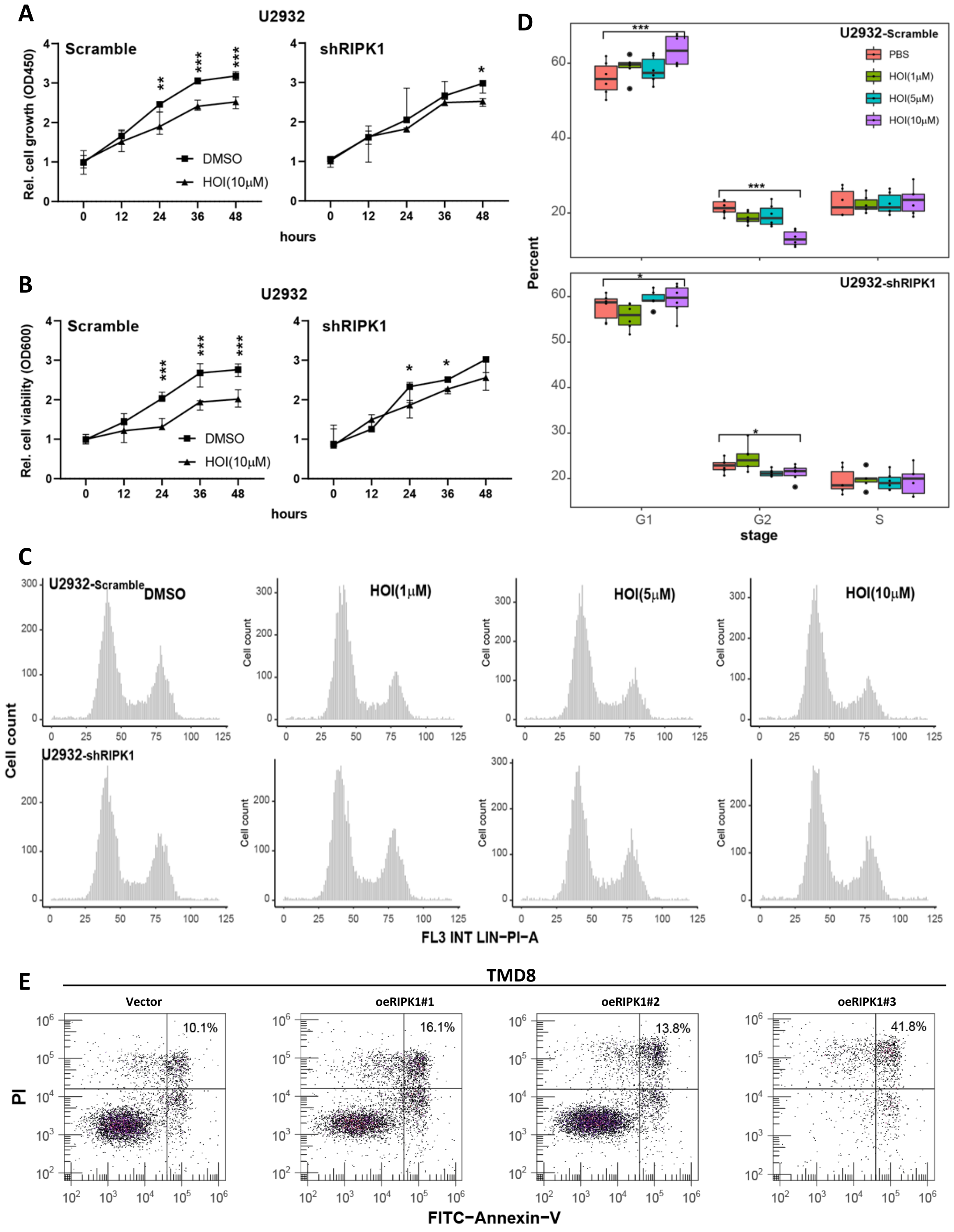

Supplement: Supplementary file 1 — Additional file 1: Fig S1. RIPK1 is down-regulated in tumor cells of lymphoma patients. A Heatmap of 3 groups of DEGs (CLL, DLBCL, and FL vs. normal, respectively). Chronic lymphocytic leukemia (CCL, N=5), follicular lymphoma (FL, N=10), and diffuse large B-cell patients (DLBCL, N=8) vs. normal donors (N=6). The cutoff was set at p-adjust < 0.001 and log2 fold change > 3. B Volcano plot of DEGs of FL vs. normal. Follicular lymphoma (FL, N=10) vs. normal donors (N=6). A cutoff of p-adjust < 0.001 and log2 fold change > 3. Fig S2. RIPK1 inhibitor promotes the proliferation of lymphoma cells A CCK8 assay determined cell proliferation of L428 lines. Cells were seeded onto 96-well plates and pre-treated with Nec (30 μM) for 24 h, and then, cell growth status was detected by CCK8 assay every 12 h. N=3, *P<0.05, **P<0.01, ***P<0.001 by two-way ANOVA. B Cell viability of L428 cell lines. Cells in A were seeded onto 96-well plates and pre-treated with Nec (30 μM) for 24 h, and then, cell growth status was detected every 12 h. N=3, *P<0.05, ***P<0.001 by two-way ANOVA. C PI staining determined the cell cycle distribution of L428 cell lines and the statistical results. ~5000 cells were pre-treated with Nec (30 μM) for 24 h and then collected for PI staining. D PI staining determined the cell cycle distribution of GM12878/L428 cell lines and the statistical results. ~5000 cells were pre-treated with RIPK1-IN-7 (IN-7, 5 nM) for 24 h and then collected for PI staining. N=6, ***P<0.001 by student’s t-test. E Annexin V-FITC/PI double-labeled FACS determined the cell death of L428 cell lines. Cells in A were used for experiments. Fig S3. HOIPIN-1 inhibits the proliferation of lymphoma cells by blocking RIPK1 function. A CCK8 assay determined cell proliferation of U2932 lines. Cells were seeded onto 96-well plates and pre-treated with HOIPIN-1 (HOI, 10 μM) for 24 h, and then, cell growth status was detected by CCK8 assay every 12 h. N=3, *P<0.05, **P<0.01, ***P<0.001 by two-way ANOVA [file 12672_2023_725_MOESM1_ESM.pdf]
